# Experiences of predictive genetic testing in inherited motor neuron disease: Findings from a qualitative interview study

**DOI:** 10.1002/jgc4.1904

**Published:** 2024-04-16

**Authors:** Jade Howard, Karen Forrest Keenan, Fadhila Mazanderani, Martin R. Turner, Louise Locock

**Affiliations:** ^1^ Sheffield Institute for Translational Neuroscience University of Sheffield Sheffield UK; ^2^ Epidemiology Group University of Aberdeen Aberdeen UK; ^3^ Science, Technology and Innovation Studies University of Edinburgh Edinburgh UK; ^4^ Nuffield Department of Clinical Neurosciences University of Oxford Oxford UK; ^5^ Health Services Research Unit University of Aberdeen Aberdeen UK

**Keywords:** amyotrophic lateral sclerosis, genetic testing, lived experience, motor neuron disease, predictive genetic testing, qualitative

## Abstract

Predictive genetic testing is increasingly available for individuals with a heightened risk of motor neuron disease (MND). However, little is known about how they decide whether or not to get tested, and how they experience this process. This paper reports findings from a constructivist grounded theory‐informed interview study with 24 family members of people with identified or suspected inherited MND (iMND). Fourteen did not know their genetic status, and nine had decided to have predictive testing, of whom six tested positive for the pathogenic gene variant identified in their family and three tested negative. One additional person was identified as negative through a parent's negative result. This paper explores the diverse ways people approached testing, and the many factors and motivations involved, based on personal attitudes and goals, experiences of living with genetic risk, and wider family considerations and circumstances. Results were met with a range of emotions; whatever the outcome, the news disrupted each person's view of the future, and they adapted in their own way and time. Support after results was variable and a perceived lack of support impacted coping and the ability to move forwards. This paper situates findings against literature on other genetic conditions, highlighting experiences as grounded in the unique characteristics of iMND. Thus, it emphasizes the need for disease‐specific guidelines and support structures around predictive genetic testing in this context. Understanding people's experiences and responding to these needs is particularly timely given the uptake of testing amongst this group is anticipated to rise with increasing access to genetic testing for people with MND, and gene‐specific clinical trials.

**What is known about this topic:**

Predictive genetic testing is a significant decision with a range of implications. However, little is known about how individuals at risk of inherited MND make such decisions and their experiences of the process.

**What this paper adds to the topic:**

This paper contributes novel insights into how individuals at risk of inherited MND came to predictive testing decisions, the factors and considerations involved, their experiences of receiving results, and post‐test support.

## 1 INTRODUCTION

Predictive genetic testing (alternatively pre‐symptomatic testing) is available in a range of conditions, enabling asymptomatic family members to find out if they are at an increased risk of developing the disease. This is the case for inherited motor neuron disease (iMND), in its most common form known as ALS (amyotrophic lateral sclerosis), an adult‐onset neurodegenerative disease, causing progressive weakness and muscle wasting. Symptoms and progression vary but may lead to a complete a loss of mobility and speech, as well as cognitive and behavioral changes. There are currently no therapies approved in the UK that offer a substantial clinical benefit to treat or cure MND. The disease is fatal within 30 months of symptom onset for 50% of individuals, mainly due to respiratory failure (Brown Jr. & Al‐Chalabi, 2017).

In 5%–20% of cases, MND is linked to a pathogenic monogenic variant. Over 25 genes have been identified, meaning a genetic cause can be identified in approximately three quarters of affected individuals with a family history of ALS, as well as a minority of people with seemingly “sporadic” disease (Roggenbuck et al., 2017; Shepheard et al., 2021). Gene variants are usually inherited in a dominant pattern, meaning children of people carrying a pathogenic variant have a 50% inheritance risk (Goutman et al., 2022), though penetrance (the chance an individual carrying a pathogenic variant will develop symptoms) is thought to vary across gene variants and families (Dharmadasa et al., 2022). Recent research suggests initial penetrance estimates for the *C9orf72* hexanucleotide expansion (the most common genetic cause of MND in people from European descent) may have been significantly inflated, adding further complexity to genetic testing decisions and genetic counseling practice (Van Wijk et al., 2023). Several variants, including *C9orf72*, are also associated with the pathologically related disorder frontotemporal dementia (FTD) and other neurological symptoms (Shatunov & Al‐Chalabi, 2021).

In 2023, genetic testing became available to all people with MND in England (NHS, 2023), having previously been guided by age and family history criteria. This change was driven in part by the success of recent clinical trials, which led to the first genetically targeted treatment being approved by the FDA. The drug, Tofersen, targets the *SOD1* gene, whose pathological variants underlie 20% of iMND cases in people with a family history of European descent, and 2% overall. It is now in trial in an asymptomatic gene variant carrier population, using rising blood neurofilament light chain levels as a “gatekeeper” marker of phenoconversion and randomization point (Biogen, 2023). Whilst these advances only benefit a subset of individuals, there are renewed hopes for success in other MND‐linked gene variants and ongoing trials.

Alongside the potential for therapy or trials and the wish to understand as much as possible about their condition, the identification of a genetic cause in a person with MND enables relatives to consider predictive genetic testing to find out if they have inherited this variant, as well as reproductive genetic testing options (Crook et al., 2021). Predictive testing guidelines are modeled around those used in other conditions, including Huntington's disease (HD) and Alzheimer's disease (Roggenbuck et al., 2017). In the UK, people considering predictive testing must provide informed consent and are generally required to see a genetic counselor at least twice before testing. There is no widely used protocol for post‐test follow‐up. A positive predictive test result confirms inheritance, but uncertainty remains over if and when symptoms will develop, and how an individual might be affected.

There are limited data on predictive testing in MND. A 2004 mixed‐methods study amongst untested, asymptomatic family members found high interest in testing, with positive attitudes based on hope of a negative result, to inform reproductive choices, alter priorities, pursue goals, and prepare psychologically. Reasons for not wanting to know included lack of preventive measures, concerns over insurance discrimination, wishing to avoid feelings of guilt, and worry about developing the condition (Fanos et al., 2004). Interviews with participants from *SOD1* families (Fanos et al., 2011) presented similar findings, with additional factors in decisions not to test grounded in a desire to maintain hope, not wanting to be overly vigilant about symptoms, uncertainty over how they would cope with a positive result, and a fear of regret. Reasons participants had opted to receive predictive test results included freedom from ambiguity, experiencing not knowing as a mental burden, to inform lifestyle decisions, provide greater certainty for children, and remove the need for children to seek testing if negative (Fanos et al., 2011).

Experiences of receiving results have also been reported in Fanos and colleagues' later study (Fanos et al., 2011), with participants receiving negative results describing relief from worries, and a resumption of their former lives, some with greater appreciation and an ability to look forwards. However, it could be hard to rethink the future, and some experienced guilt (though this often lessened over time). Responses to a positive result included feeling frantic, devastated, and distressed, though most came to terms with the result relatively quickly, and reported positive changes, including motivation to take care of health, clearer priorities and perspectives, and changed life decisions. Nonetheless, some found that concerns over developing the disease escalated over time. It is worth noting that these studies took place in the United States, before the discovery of the *C9orf72* gene expansion, which was transformational in enabling more families to understand the genetic cause of the disease and seek predictive and reproductive genetic testing. Recent research amongst individuals who tested positive for MND/FTD‐linked gene variants highlights that people experienced a threat to self, identity, and ability to fulfill future roles as a result of this knowledge (Dratch et al., 2023).

There has been considerable research on genetic testing in other conditions, including HD and hereditary cancers. However, in hereditary cancers, there may be screening and prophylactic surgery options to reduce risk and enable early detection, as well as treatment options for those diagnosed. Whilst HD lacks screening and treatment, it is a fully penetrant condition (with some exceptions), increasing the certainty and potential utility of genetic test results (Hartzfeld et al., 2015). Thus, the distinct disease characteristics, treatment options, and genetic architecture of iMND introduce specific uncertainties and complexities, underscoring the need for research amongst this population (Crook et al., 2021; Roggenbuck et al., 2017). This is especially pertinent given ongoing gene‐specific clinical trials and widening of eligibility criteria for genetic testing for people with MND (NHS, 2023), which may lead to more relatives considering predictive testing (De Oliveira et al., 2023) and requiring genetic counseling (Dratch et al., 2023). There is therefore a growing urgency to understand attitudes and experiences around predictive testing and ensure adequate support is available (McNeill et al., 2022).

Our study responds to this need through exploring how people at risk of iMND come to genetic test decisions, the factors and considerations involved, their experiences of receiving results, and of post‐test support.

## 2 METHODS

### 2.1 Participants

This paper draws on a wider interview study on the experiences of families affected by iMND, which also included people with inherited forms of MND and partners. It was guided by an interpretivist approach and constructivist grounded theory‐informed methodology (Charmaz, 2014). The authors draw on diverse experiences, with backgrounds in social science and health services research (JH, KFK, FM, and LL), and neurology (MRT). This paper reports data from a subset of participants: 24 unaffected family members of people with suspected or confirmed iMND, of which nine had had predictive testing (with six testing positive and three negative). One additional person had been identified as negative through a parent's negative result. The remaining 14 participants did not know their personal genetic status but understood themselves to be at an increased risk of developing MND due to their family history. In some families, the gene variant responsible for the disease was not known, in spite of a strong family history, meaning predictive testing would not be recommended. Participants ranged from their 20s to 60s, were based in the UK, and all but one identified as white (British/other white backgrounds). Participant characteristics are detailed in Table 1. Pseudonyms and age ranges have been used to protect participants' identities. One participant dropped out following the interview for unknown reasons; these data have not been included.

**TABLE 1:** Participant characteristics.

Pseudonym	Sex	Age	Gene variant in the family	Predictive testing status (time since results)	Children
Abigail	F	40–49	*SOD1*	Not tested	No
Aileen	F	50–59	*C9orf72*	Not tested	Yes
Amelia	F	30–39	*C9orf72*	Not tested – confirmed negative through parent's genetic test (3 months)	No
Amy	F	20–29	*C9orf72*	Tested – negative (4 months)	No
Becky	F	30–39	*C9orf72*	Tested – negative (4 months)	No
Cath	F	50–59	*C9orf72*	Tested – positive (7.5 years)	Yes
Dave	M	40–49	Known but cannot remember	Not tested	Yes
Eilidh	F	30–39	*SOD1*	Not tested	Yes
Emma	F	30–39	*C9orf72*	Not tested	Yes
George	M	20–29	*C9orf72*	Not tested – undergoing genetic counseling	No
Helena	F	60–69	*C9orf72*	Tested – negative (6 weeks)	No
Henry	M	40–49	*C9orf72*	Tested – positive (2 years)	Yes
Jack	M	20–29	Unknown	Not tested	No
Jake	M	20–29	*C9orf72*	Tested – positive (5 months)	No
Jan	F	50–59	Unknown	Not tested	Yes
Jess	F	30–39	*C9orf72*	Tested – Positive (8 months)	Yes
Lisa	F	30–39	*C9orf72*	Tested – positive (15 months)	Yes
Mae	F	50–59	*C9orf72*	Tested – positive (15 months)	No
Mike	M	40–49	*C9orf72*	Not tested	Yes
Miriam	F	50–59	Unknown	Not tested	Yes
Sam	Withheld	Withheld	*C9orf72*	Not tested – Undergoing genetic counseling	Withheld
Sandra	F	40–49	Unknown	Not tested	Yes
Sophia	F	20–29	*C9orf72*	Not tested	No
Sorley	M	40–49	Unknown	Not tested	Yes

### 2.2 Instrumentation and procedures

Participants were recruited using a maximum variation sampling approach (Patton, 1990) through multiple avenues, principally the MND Association and MND Scotland (including mailing lists and social media), a research network for families affected by iMND, and snowball sampling. Following expressions of interest, participants were contacted by their preferred method (phone, email). Plans to recruit through the NHS were hampered by the COVID‐19 pandemic. Recruitment continued until the interviews were deemed to hold sufficient “information power” (Malterud et al., 2016, p. 1759) and the research was deemed to offer “new insights that contribute substantially to or challenge current understandings.”

Individual interviews were conducted by a female researcher (JH), both face‐to‐face (principally in people's homes) and virtually (during the pandemic), with consent taken following a pre‐interview meeting. Interviews used an intensive interviewing approach (Charmaz, 2014), with an evolving topic guide used flexibly to guide discussion. This was informed by literature, a study of online forum posts (Howard et al., 2021), and feedback from family advisors and a wider advisory panel. Interviews were audio‐ or video‐recorded in line with participant preferences and used to develop a resource on iMND on the healthtalk.org website (https://healthtalk.org/introduction/inherited‐motor‐neurone‐disease‐mnd/). Interviews lasted an average of just under 2 h. One participant was interviewed a second time after finding out her genetic test result.

The potential for participant distress was carefully considered. Where participants became upset, the researcher acknowledged distress, allowed them to express their emotions, and decide whether and when to continue (Braun & Clarke, 2013). All participants opted to continue, with the sequencing of questions aiming to bring discussion back to a “normal” rhythm before finishing the interview (Charmaz, 2014). Participants were offered a list of support services/resources where appropriate.

Given the emotion work (Dickson‐Swift et al., 2009) that can be involved in doing research with people who have faced distress, JH had frequent supervision to talk through any issues raised with her supervisors, who had experience of interviewing individuals affected by genetic and life‐limiting conditions.

### 2.3 Data analysis

Audio recordings were transcribed professionally and checked transcripts returned to participants for revisions and approval. Transcripts were coded by JH using a constructivist grounded theory‐informed approach (Charmaz, 2014), facilitated by NVivo. Here, inductive coding was developed into focused coding, which involved interrogating and comparing the codes generated to develop a thematic framework, with memos recorded throughout data collection and analysis. Each coding report was then analyzed further to examine the breadth of data in each code and evolve the analysis to a more conceptual level (Ziebland & McPherson, 2006). Developing topic summaries were discussed with family advisors and the wider advisory panel.

## 3 RESULTS

This paper presents results from the theme predictive genetic testing. This was a central theme in the wider study, and one of 12 themes developed on diverse aspects including living with inherited forms of MND; living with genetic risk; knowledge, information and support; and reproductive choices. Subthemes around predictive testing were developed to explore key dimensions of participant experiences and are used to structure results below.

### 3.1 Coming to a decision

People came to genetic testing decisions in a variety of ways. Some described an immediate and self‐evident understanding where they “just knew” whether they wanted to have the genetic test or not, expressing little ambivalence or uncertainty. Mae recalled her reaction to finding out about the *C9orf72* gene expansion in her family and the possibility of predictive testing: “I don't think I paused for a millisecond, I wanted to know… I didn't waver from that thought… it was immediate.”

Others described weighing up factors and implications, with some moving linearly to a decision and others shifting between options, at times expressing ambivalence. Jake began thinking about predictive testing after finding out about his risk of MND through his parent's diagnosis. He worried initially about financial and professional implications, yet over the next 18 months decided the benefits outweighed the drawbacks: “You're constantly weighing it up every week, like, ‘Why, what do I get out of it? What's the risk of knowing?’ It just came to a point where I was like, ‘Look, I need to know’.” Whilst some, like Jake, proceeded to have the test, others remained unsure of their decision. Aileen had times when she thought she might like to know, “but then I'll talk to people… and most people say, ‘No, don't’.”

Whilst taking the test is an irreversible decision, the choice not to have it can be revisited. As Dave explained: “This is a continuous decision. It only ceases to be a decision when I have the testing and I get a result.” Whilst Jan could not imagine any circumstances where she would want to be tested, a more common approach was a suspended decision, with an openness toward being tested in the future. Emma said, “I'm not going to get tested now, but… I have absolutely no idea whether or not I'll get tested in my lifetime.” Here, testing was seen to be of little benefit given the lack of (preventive) treatments, but individuals felt they might reconsider as trials and treatments advanced. Another priority was the benefit for the wider family, with participants suggesting they might reconsider if testing could provide useful information for children. Decisions were also grounded in perceptions over ability to cope with the information. Eilidh thought she might reconsider if she was “in a better place mentally.”

### 3.2 Factors and considerations

#### 3.2.1 Deciding to have the predictive genetic test

Some people found it difficult to live with uncertainty. Jake believed that spending the next 25 years questioning his genetic status would be worse than a positive result. Cath similarly wanted a sense of certainty and control: “I was living in this ‘Am I, aren't I’… I was living in limbo.” Mae's decision was grounded in the fact she was already worried by her 50% chance of inheriting the *C9orf72* gene expansion: “I couldn't see the difference in that knowledge, it wouldn't give me any more peace to have not had the test and sit there thinking, ‘It's 50/50’; you've still got that chance of being affected any day.” For Henry, the question of whether to be tested was *itself* a source of “emotional stress.” He felt he would be better off knowing he had inherited the MND‐linked gene than not knowing, so he could “move past that decision.”

It could also be difficult to deal with worry over possible symptoms. Helena recalled times where she had convinced herself she was showing signs of the disease and questioned whether she was worrying unnecessarily. Others also emphasized the hope of receiving a negative result. Whilst “thinking gene‐positive” (Duncan et al., 2007) was widely described in interviews, Amy, by contrast, had a “gut instinct” she would test negative, which made her less fearful of having the test. Having lost her mother and aunt, her view was that she had little more to lose: “What am I really losing? I lived for my mum, and you've taken her, so it is what it is, really, whatever my result is.”

Genetic testing was about being prepared and planning ahead. Whilst not everyone felt MND risk was relevant in reproductive decisions, some saw testing as offering information to inform their choices. Lisa found out about iMND in her family soon after her son was born. She and her husband felt they needed to know if she carried the *C9orf72* gene expansion before having another child: “We weren't prepared to continue with our family until we knew the outcome.” Even though they did not imminently plan to have children, Jake and Becky did not want their children to go through the “trauma” of knowing they were at risk of developing MND. Jake saw stopping the disease as a “no brainer”: “I didn't want to continue a path down a life and build a life for me that could be destroyed by motor neuron disease. I'd much rather be able to build a family and protect it.” Becky described not knowing her genetic status as an inability to move forward and found it helpful to prepare for the possibility of both positive and negative results so whatever happened, she could progress with having a family: “I was at a crossroads and I had two choices, two roads, and they were both on a red light. And the road I was choosing was not down to me. So, it was easier to focus on the steps on each route that I could work to.”

Having predictive testing was also about having concrete information for children, to inform communication and enable them to make informed choices around having families of their own. As Cath said, “They've got far more solid information than I ever did, and I wanted that for them.” Henry felt that not knowing, he would have been “in even more of a quandary” about what to tell his children, whereas following a positive result he could then plan *how* to talk to them.

Sam, who was beginning the genetic counseling process, felt a positive result would motivate them to consider lifestyle changes that could delay symptoms, as well as options around treatment, care, and end of life. People also felt knowing their genetic status could inform financial planning, and practical choices such as housing. Having seen his father struggle as his disease progressed, Jake wanted to “plan for my future to be disabled friendly.” People also felt this knowledge could inform how they lived their lives in the present, including getting involved in fundraising and research.

Participants felt that predictive testing would make them better prepared if they were to develop symptoms, leading to an earlier diagnosis and access to trials and treatments. Helena's brother had considered taking part in a clinical trial, but his symptoms were too advanced. She felt “being forewarned might be a good thing.”

#### 3.2.2 Continuing life without having the predictive genetic test

Deciding to continue without predictive testing was an equally multi‐faceted choice. Whilst receiving a negative result would be “amazing,” “phenomenal,” and “life changing,” the possibility of testing positive was of concern, and for some, seen as too high a risk. Gambling metaphors were used to compare taking the genetic test to tossing a coin, rolling the dice, or playing “Russian roulette.” Emma said: “Obviously if I found out I didn't have the gene, that would be great, but you have to roll the dice, don't you?” Sophia saw taking the test as “such a risk for me. As it's not affecting me, it's just not worth it. But if it was, it probably would be.”

Not having the genetic test was also about maintaining hope of not having inherited the gene variant. Aileen explained: “I'm convinced I've got it anyway. But I'm not 100% convinced, I can be 70% or 80% convinced, and that 20%/30% is the difference I think between me being very depressed and preoccupied than not being… not knowing for sure allows me to have hope.”

Another argument was that knowing what could happen in the future would be “pretty grim.” Jan said, “Why do I want to know if there's a bomb going to go off?” People were concerned about the potential emotional and mental health impact of a positive result and felt they could cope better not knowing. Jack said: “If I get told I've got it, in my mind, days are numbered in that sense… personally it would mess with us.” Emma was unsure if she would be able to “pull myself back” from a positive result, and Sorley was uncertain of how he would react: “At the moment you're talking about an untreatable, fatal condition… what effect would that have on somebody, knowing that that's going to happen? … I don't really want to find out, to be honest.” Another fear was that knowing for sure would increase worries over possible symptoms. Miriam's concern was that she would be “even more anxious and more hypervigilant, and I'm bad enough as it is.” Aileen highlighted the irreversibility of the knowledge gained from the test: “If I knew that I had the faulty gene, there's no way back from that.”

For people who had worked to position their thoughts and worries around iMND in a manageable place, there was a concern that engaging with testing could unsettle coping strategies and bring worries to the forefront. Having got into a positive mindset, Mike wanted to avoid revisiting the decision.

Some people considered the impact on family members if they were to seek testing. Amelia had a “gut instinct” that she would want to be tested, but did not engage with the decision whilst her mother, Helena, did not want to know; her result, if positive, would mean Helena also carried the *C9orf72* gene expansion: “Me going and getting tested ahead of my mum was taking that decision away from her and that just didn't seem fair at all.” Other people raised the implications for children; testing positive would confirm their children had a 50% chance of inheritance. Knowing her daughter did not want to be told her test result was one of several factors Aileen mentioned when discussing her choice not to find out her genetic status.

Concerns also centered on financial implications of a positive result, including on insurance or securing a mortgage. Jack had come across a question on a life insurance form about medical history: “I'd rather not know because the minute you put something like that down, they're just going to ram your premium straight up.”

Some people felt predictive testing would not provide useful information, particularly given the current lack of treatment to prevent, halt, or cure the disease. Sophia explained: “If I knew I had it, I would worry about it even more and there was still nothing I could do about it… It would only change my life in a negative way.” Family context, stage of life, and personal goals set the backdrop against which the utility of testing was assessed. For example, Dave might have considered testing if he had not already had children when he found out about iMND in the family.

Perceiving test information to have limited value was also based in part on remaining uncertainties for people testing positive. Participants pointed out that even if this happened, they might never develop symptoms, and would not know when symptoms would develop or what they would be. Sorley summed up this view: “I wouldn't anticipate getting an easy to deal with, black and white answer.”

Several felt knowing their genetic status would not change how they lived. Aileen chose to behave like she had inherited the gene variant anyway, and Dave's “philosophy” was that the future is uncontrollable for many reasons. Sandra, by contrast, felt that having the test would change how she lived her life – “Is that a good thing or a bad thing? I'm not quite sure.”

#### 3.2.3 The role of others in the decision

Whilst participants described having open and honest conversations about inherited MND and genetic testing, in other families, it was a “taboo subject”, and relatives were involved to a greater or lesser extent. Henry was one person who felt it was important to involve his wife in discussions, since “you both live with that disease.” Mae talked to her partner about her decision but “didn't ask an opinion.” One person's partner was opposed to her having the test, so she went ahead without telling him. For some, talking to family members raised new considerations and shifted their approaches. Siblings and cousins were sometimes valued as people to talk to, as they were often in a similar situation. People struggled when they felt others dismissed their worries or could not understand.

People generally sought a referral to clinical genetics when interested in having the predictive test. Lisa had an appointment with her mother's consultant, but other participants had two or more sessions with genetics professionals before the test. Whilst experiences varied, genetic counseling was generally seen as helpful in providing information on MND and areas such as reproductive options, and people appreciated empathetic support. However, frustrations were expressed at waiting times between appointments and the requirement for multiple sessions. Jake and Henry described a sense of being “assessed,” and felt they had to be firm in their reasoning in order to have the test. Becky faced an additional barrier as her father did not want to know his genetic status, and her genetic counselors asked her to give him time to make a decision. She described “fighting” for the test, which was carried out after delays, and felt that she should not have been impacted by his (non)decision: “I wasn't being allowed to make decisions about my own health.”

Two people went to a neurologist for advice and felt they were discouraged from predictive testing. Both reported finding these conversations helpful and neither saw a genetic counselor or pursued testing further. Another person described being told by their GP that having a predictive test would not be of benefit and would cause more worry. Although she felt “dismissed,” she thought her GP was “probably right.”

### 3.3 Receiving predictive testing results

#### 3.3.1 Receiving a positive result

Participants described receiving their test results. Jess was told over the phone due to the COVID‐19 pandemic and recalled her experience as “a bit of a blur” where “my world just stopped spinning for a moment.” Others felt “empty” or “frozen.” Mae did not get upset until after the appointment but described “an overwhelming desire just to get out of there.”

Even where people expected a positive result, it was shocking and disappointing to be given the news. Henry said, “Even though you go in there and you're dead set to have it… you're still deflated when you're told.” Jake felt “gutted” and struggled to comprehend it: “It didn't sink in, nobody asked me a question about it, they were just shocked for me, but everyone else's life just goes on like normal whereas your life doesn't.” Cath similarly recalled difficulty processing the result, which could be challenging for partners too: “I remember opening it and feeling a little bit anticlimactic… I don't think I absorbed the information very well that day, and I don't think my husband did either… it was a very surreal day.” Jess, however, found the threat of the disease became real: “It made something that was a possibility become real, and in no way like anything I've ever experienced before…At that point, it felt like a death sentence.” Lisa had recently lost her mother, whom she had cared for, and immediately felt the implications for her own future: “My brain went to… I'm going to go through everything my mum has just gone through… my brain jumped a few decades.”

The implications of the results, both long term and short term, made them particularly hard to take. For Lisa, it complicated her immediate hope of expanding her family and brought decisions around reproductive genetic testing: “It changed the future, and the immediate future. Life became difficult straightaway, which was disappointing.”

The implications for existing children were also on people's minds. Jess was pregnant when she found out about her risk of MND, and received a positive predictive test result after having her daughter: “The weight of the guilt for having my daughter was overwhelming. I was like, ‘…what have I done?’ That was hard.”

People processed and moved forwards from the test results in their own way and time. Although relieved to know his result, for Jake finding out he carries the *C9orf72* gene expansion has been “another weight on your shoulders.” He found this difficult to process, particularly as he did not feel there was anyone he could talk to who understood how he felt. Although he has ambitions, Jake described times where he found it hard to see the point of carrying on. This has been made harder by ongoing uncertainties around if and when he might develop symptoms.

Cath found knowing much harder than anticipated, and described trying to end her life four or five times, which also affected her children's wellbeing. Having family counseling privately in the years that followed helped her understand her result as a form of loss and find a turning point: “I'd been given news that equated to loss, and no signposting and no direction of what to do with that… it gave me a loss of what might be, it gave me a loss of maybe being a grandmother perhaps, it gave me a loss of growing old and grumpy with my husband perhaps, it gave me a loss of some of my own sense of self.”

Others suggested they were able to move forwards with their lives relatively quickly. Though she felt her outlook had changed, Jess' feeling of receiving a “death sentence” gave way to a more moderate view: “Once you have time to process that information and process the implications… you kind of just get on with it.” Henry found the initial upset only lasted a few days: “After the initial shock, then you have to go back to why you got the test in the first place.” Like Jess, he tried to focus on living life to the full, rather than dwelling on something that “may or may not happen.” This uncertainty and ambiguity of something that “may or may not happen” sometimes made it difficult to know who to tell and what. Mae had not shared her results beyond close friends and family as she did not want people to pity her or make allowances: “I have told a very few of my friends, but not all of them, because on the face of it there isn't anything wrong with me.”

Results were received in the wider family context. It could be upsetting to hear a family member carried a gene variant linked to iMND, yet it could also be emotional to hear that a family member tested negative, particularly if the person hearing the news had themselves tested positive. Participants described feeling upset, jealous, or alone, even if the feeling passed quickly and they felt guilty afterward. One person explained: “I'm not proud of this feeling – but couldn't help but feel like…. ‘Oh, I'm out here on my own with this’. And it's not for a minute wanting them to be positive, but there's that ugly emotion bitterness slightly with it.”

Another individual described how communication with their sibling had changed since they had tested positive and their sibling negative; they felt their sibling could not understand and put less effort into looking into the disease and research developments. Another participant was the only one of her siblings and cousins to test positive, and had a sense of “taking one for the team.” She felt she could cope and said, “If it had to be one of us, I'd rather it be me.”

In spite of their feelings at the time and the challenges they had experienced since, no one reported regretting having the test. Whilst not the outcome they had hoped for, a positive result appeared to give people the knowledge they had sought. Mae felt she was no more worried than she was before the test – for her, “It's a different kind of worry.” However, it is worth noting that the impact of risk and preoccupation with the disease appeared to fluctuate over time and with changing circumstances (Howard et al., 2024).

#### 3.3.2 Receiving a negative result

People experienced a range of reactions to being told “good news,” including shock, relief, gratitude, and a sense of luck or having “escaped something.” Becky had a sense of a weight lifting from her, and it felt “surreal”: “I was so convinced that that was going to come back and say positive, I just sat there and cried. Then I started laughing and I was just like so thankful, and I was just kind of shell shocked.”

Helena had not allowed herself to hope that she would receive a negative result. Given that her siblings who had been tested all carried the gene variant, she felt “absolutely totally taken aback” when told she did not. Having worried about the implications for her children and grandchild and put a lot of thought into how she would share the news of a positive result, “It was just, ‘Oh, I don't have to do any of that’ and that was a very, very good feeling.” Amelia found out she had not inherited the *C9orf72* gene expansion through her mother's, negative result. She felt disbelief, and a sense of gratitude: “I said ‘Thank you’ to the universe or something, I was just like, ‘Thank you. I don't know who you are that I'm talking to, but just thank you that we've been spared’.”

At the same time, receiving a negative result could be “bittersweet” where family members had tested positive or had not been tested, and some felt anxious about sharing their results with them. Amy's cousin's positive result shaped her feelings toward her own: “My partner actually says to me a lot, ‘How do you feel?’ … and honestly, I was just like, ‘I feel fine’. But I think that is because of my cousin's results. I'd probably be screaming it from the roof if it wasn't the case for her, because I'd be like, ‘We're done and dusted with this disease…’ but that's not the case.”

Coming to terms with a negative result was an individual process. Helena found once she had the good news, she needed little time to process it: “It was almost as though there were two boxes, one half open and one properly shut, and the half open one disappeared, and the shut one just burst open and it was entire… It's not an anti‐climax, because that would be completely ridiculous, but from having so much to think about, so much to worry about and discuss…it was just done.” Amelia found adjusting less straightforward: “You kind of come to the end of the road, but you've been on this road for a really long time… there's all these thoughts and feelings and fears and emotions…they don't just dissipate… there's this feeling of not quite knowing what to do with it all and where to put it.” She found it strange to continue with her everyday routines in the context of this news: “The world keeps turning and you get up each day and there's stuff to do and you've got to go work, but you almost feel like, ‘hang on a second, how can I just be going about my day to day business, how can I just be making tea when we've got this huge news, it's so big?’”

For some people, a negative result was an adjustment to the future they had imagined. MND had felt close for Helena, who had kept her brother's mobility equipment after he died and been considering if she would need to move house to somewhere suitable for adapting. Having worried about being a “burden” on her family, her result enabled her to return to her former identity, including as a parent, “I didn't realise I wasn't me until I was me again.” She described a renewed sense of possibility and future: “All this past two years, long term really hasn't happened…. Now suddenly, the horizons have opened up a bit more.”

For younger people, adjusting to the result enabled them to move forwards with plans and milestones. Becky said: “We are just extremely lucky that we get to go ahead with our future the way we wanted to. We will still be looking at adopting or fostering in the future as well as having our own kids… we're applying for our mortgage, he's [husband] setting his business up… we feel like we've got a lot more opportunity open to us now.” Amelia felt grateful to be able to consider having a family without additional testing: “I can just think about having children when I want to, when it's right for me, with all the normal considerations… talk of IVF and embryonic testing, all of that just falls away.”

Some felt an appreciation for health and life in general. Becky gave up smoking on receiving her results: “It kind of changed my viewpoint on a lot of things… I've got a relatively healthy body and I'm going to do what I can to keep it that way… you realise how precious it is when you run that risk of losing it.” Helena had become less conscious of her body since her negative result; before the test, she had had periods of worrying about possible symptoms, but now, “It's just nice… to not have to worry about every time you walk down the road and you trip.”

These individuals felt pleased with their decision to get tested; Helena and Becky felt glad they had waited until the time was right. However, all participants pointed out that MND would continue to be part of their lives; they had lost relatives to MND, had others currently affected, or were worried about family members at risk.

### 3.4 Post‐test support

Support following predictive test results was variable. Some people received phone or email contact from their genetic counselor in the weeks that followed, and in one case a year later. Participants were told they could contact their genetic counselor if they needed anything or had questions. However, others found that finding information and support in this period was “off your own bat.”

Mae saw her genetic counselor after receiving the results, but did not feel that he was well placed to support her: “We kind of sat in the room staring at each other because he isn't there for managing sleep and anxiety.” He suggested she ask her GP to refer her for Cognitive Behavioral Therapy (CBT) and told her about opportunities for research participation and an information day, though these suggestions seemed like “after‐thoughts rather than a plan.” She felt there was insufficient follow‐up: “I went into a dip after the result because I just felt totally unsupported… that was the worst bit of it… it sent my head almost exploding that one of the ladies at [meeting] said, ‘Well I was just speaking to my neurologist the other day’, and I thought, ‘How come you've got a neurologist?. Who's supposed to tell me where to do that?’ So that has been my bugbear, is that you're all at sea.”

Whilst not everyone felt they needed post‐test follow‐up, others similarly described a lack of adequate support and a sense of being “abandoned.” Participants juxtaposed this with the support they received in the process of having the test. Cath felt angry about this and believed there should be at least 6 months of follow‐up, and Jess suggested tailored counseling should be “mandatory” for those receiving a positive result. Other suggestions included having a dedicated person to routinely check in with the individual and family after a positive result, opportunities to hear from others in a similar situation, standard information sheets covering next steps including where to find out about participating in research, and ongoing monitoring to assess health. After his result, Jake asked his GP to see him for annual monitoring.

Participants who had received negative results described limited follow‐up. Whilst not everyone felt they needed it, individuals expressed a desire to get involved in research and awareness raising, and for information on supporting family members.

## 4 DISCUSSION

This paper explores participant experiences of predictive genetic testing, highlighting the pathways people took in coming to decisions, and the multiple and intertwined factors and considerations involved, based on personal attitudes and goals, experiences of living with risk, and wider family considerations and circumstances. Results were met with a range of emotions, but whatever the outcome, this news often disrupted each person's view of the future and they processed it in their own time. Support after results was variable and for certain individuals with a positive result, a perceived lack of support impacted coping and ability to move forwards.

Findings highlight the multiple approaches people took in coming to choices around predictive testing, reflecting literature that categorizes decision‐making into overlapping pathways (Cox, 2003; Etchegary, 2006). A key point is that what is often framed as a “decision” is not always approached as a choice. For some, “deciding” to get tested was an immediate response, with little ambivalence or conscious reflection. However, those that chose not to get tested generally described this as a decision not to have the test *now*, with the majority conceiving of a future where they might consider testing, grounded in shifting personal and familial circumstances and an evolving research landscape. Similar experiences have been reported by those at risk of HD (Taylor, 2005). Our findings thus reinforce that predictive testing could increase if hopes for (preventive) treatments are realized (De Oliveira et al., 2023).

Participants expressed diverse factors, motivations, and considerations in predictive testing decisions, grounded in experiences of living with uncertainty, (perceived) ability to cope, and the utility of results for themselves and their family. Research on HD similarly highlights intertwined personal and familial factors involved in testing decisions. Genetic testing was sought in order to seek certainty over inheritance and overcome the anxiety of not knowing; shape decisions and future planning (e.g., reproductive, career, and financial planning); seek information for children; and prepare for the possibility of developing the condition (Chapman, 2002; Cox, 2003; Etchegary, 2006; Konrad, 2003; Leontini, 2006; Richards, 2004; Taylor, 2004). Deciding not to test was broadly linked to a desire to preserve hope and keep risk at bay (including for children) (Konrad, 2003; Quaid et al., 2008); the perceived need for the test; and possible impact of a positive result (Etchegary, 2006; Forrest Keenan et al., 2015; Richards, 2004; Rivera‐Navarro et al., 2015; Taylor, 2004, 2005). The concept of genetic responsibility (Hallowell, 1999) elucidates the above findings. As Cox (2003) points out, genetic testing decisions are constrained by social and familial ties, which limit and give meaning to the range of acceptable choices. We highlight diverse approaches to communication in families and illustrate how (divergent) views and experiences around genetic testing can impact communication and relationships.

Receiving a positive result could be shocking and devastating, with participants describing worries over developing symptoms, sadness over the loss of an anticipated future, guilt and fear for children, and increased self‐monitoring. Huntington's literature points to similarly negative impacts, but like our paper also emphasizes coping and adjustment, with some feeling able to put the result to the back of their minds, use the information to move forwards and to inform plans. In our research and existing literature, participants described changed priorities and perspectives towards the important things in life (Chapman, 2002; Duncan et al., 2007; Forrest Keenan et al., 2015; Gong et al., 2016; Hagberg et al., 2011; Leontini, 2006). Implicit in interviews was a heightened awareness of time. Participants described life feeling shortened as the future – although uncertain – came into sharpened focus. Finkler (2005) describes the collapsing of past and future in genetic testing, where “we get ‘instant messaging’ from the past and glimpses of future time.” It is notable that no one in our study reported regretting their decision.

Whilst receiving a negative result was met with relief and gratitude, this study reflects previous research in suggesting people experience both losses and gains as they adapt to this knowledge, leaving behind past worries and an identity as a person “at risk” (Cox & McKellin, 1999; Forrest Keenan et al., 2015; Winnberg et al., 2018). Whilst participants had started to plan toward developing the disease, they generally appeared to go back to plans they had before becoming aware of their risk.

As discussed, decision‐making around predictive testing and responses to results have similarities with those reported in other incurable inherited conditions, especially HD. However, the characteristics of iMND resulted in a distinctive and shifting terrain of uncertainty. Here, who or what was “at risk” evolved through the testing process. In deciding whether to have the test, what seemed to be “at risk” for many was the hope that they might not have the MND‐linked gene. A negative test result, although closing the box on a kind of “embodied risk” (Kavanagh & Broom, 1998), did not remove the impacts of the disease in the family and uncertainty related to other relatives' risk. Furthermore, participants described immediate and longer‐term uncertainties in self‐understanding, with ambivalence around communication. A positive test result seemed to consolidate and dramatically reinforce people's status of being “at risk,” but there was still uncertainly, shifted toward when they might get the disease, what symptoms (or condition) could present, and the rapidly shifting research landscape, bringing hope for treatments “in time.” A key distinction from HD, however, was the cushioning hope that it might not manifest at all. Interestingly, hope was less prevalent in Dratch et al.s' (2023) study with people who had tested positive for MND/FTD‐linked gene variants. Here, uncertainty centered more on “anticipated decline on an uncertain timeline” (Dratch et al., 2023, p. 7), leading the authors to call for further research into the role of reduced penetrance in narratives of hope.

### 4.1 Research and practice implications

This study suggests that genetic counselors may be valued as a source of information and support, more than as facilitators in decision‐making, reinforcing findings from MacLeod and colleagues (2014) that most people who had had predictive testing seemed to have decided to have the test at the point of seeking a referral to genetic counseling. Nonetheless, genetic counselors are trained to support people to explore their options and make an informed decision. Therefore, people should be referred for genetic counseling at the point of expressing interest in predictive testing. Additionally, interviews have highlighted multiple areas where participants required additional information, including the timelines for the testing process, how results are presented, and the legal requirements for disclosing test results. Genetic counselors would be well placed to respond to these needs and should consider providing information in a range of formats that can be revisited.

Generally, it is important for healthcare professionals to keep in mind that predictive testing often happens in a period of existing stress (Crook et al., 2022), alongside the diagnosis and deterioration of the disease in a relative, or in the context of grief and bereavement. Thus, our findings reinforce research on HD, which highlights that there may be a gap in support that takes into account this context of existing stress and loss (Forrest Keenan et al., 2015).

Given the significant emotional and practical implications of predictive testing, people require adequate support to meet their needs and facilitate adjustment, which is particularly challenging in the context of ongoing uncertainty post the test result (Biesecker & Erby, 2008). Previous research on predictive testing in iMND has suggested that emotional upset after receiving results is temporary, with Fanos et al. (2011) reporting that thoughts about suicide were “not dwelled upon but part of a myriad of other intense but transient emotional responses.” This study, however, suggests that a positive result and lack of support afterward can contribute to serious mental health implications (including suicide attempts). It is worth noting that HD research may have underestimated the impact of a positive result (Etchegary, 2011). Timman et al. (2004) reported that hopelessness amongst people testing positive increased in the 7‐ to 10‐year follow‐up of their study – concluding that “testing for fatal inherited diseases creates a long‐term, lifelong stress.” As outlined in the context of HD (Etchegary, 2011), understanding risk as “chronic” (Kenen et al., 2003) has implications for follow‐up. This study evidences a dearth of support for people receiving positive results and reinforces the need for expanded and ongoing support. A family‐systems‐based therapeutic approach (Rolland & Williams, 2005) has elsewhere been suggested as appropriate for this population (Dratch et al., 2023), given that the social and psychological implications of living with a genetic condition are experienced in a family context.

Receiving a negative result brought challenges such as guilt and worry over sharing the news. Whilst participants had differing support needs, research shows that group support can help people adapt to a negative result and feel less isolated (MacLeod et al., 2018). The challenges of adapting to news of non‐inheritance for one individual who found this out through a parent's genetic test underscore the need for support for family members who have not had genetic testing themselves. This is reinforced by the fact that living with genetic risk can have significant emotional and psychological impacts regardless of whether a genetic cause of the disease has been identified in the family or confirmed in the individual (Howard et al., 2024). A future research priority should be in establishing an evidence base around what forms of support would be acceptable and beneficial to individuals with a variety of genetic test decisions and outcomes. For this to reach all who need it, options both within and outside of genetic counseling contexts could be explored.

Interviews included individuals at different life stages and circumstances, offering broad insights into living with risk. However, some experiences and aspects were covered minimally. For example, research with people who had used reproductive genetic testing could elucidate a wider range of experiences. Whilst participants in this research generally came to predictive testing decisions with knowledge of the disease and it's progression based on their family history (at times over generations), the widening eligibility criteria for MND genetic testing (NHS, 2023) mean more people with assumed “sporadic” disease will find out they have an inherited form, thus highlighting the risk to their relatives. This may impact their predictive testing decision‐making and information and support needs, warranting further research amongst this subset of individuals.

The above findings suggest that perspectives were temporally grounded; had people been interviewed at a different point they may have prioritized different aspects. A longitudinal approach could be used in future research to explore the “chronic risk trajectory” (Kenen et al., 2003). Furthermore, for those with a negative result, the short time between the result and the interview raises questions on how experiences might change with long‐standing knowledge. Whilst this paper focused on genetic testing in the context of everyday lives, future research could attend to how testing decisions are negotiated in clinical genetics, thus building on extant literature which has interrogated how risk identities are (re)constructed in genetic counseling consultations (Armstrong et al., 1998).

## 5 CONCLUSIONS

This study explores experiences of predictive testing amongst individuals at risk of iMND, contributing novel insights into how people came to test decisions, factors and considerations involved, experiences of receiving results, and post‐test support. In doing so, it responds to a need to explore the lived experience of MND predictive testing in the context of developments in genetic knowledge and technologies and an evolving research landscape.

## AUTHOR CONTRIBUTIONS

JH and LL confirm that they had full access to all the data in the study and take responsibility for the integrity of the data and the accuracy of the data analysis. All of the authors gave final approval of this version to be published and agree to be accountable for all aspects of the work in ensuring that questions related to the accuracy or integrity of any part of the work are appropriately investigated and resolved.

## CONFLICT OF INTEREST STATEMENT

LL declares that she became a member of the MND Association's Health Research Advisory Panel in 2021. JH, KFK, FM, and MRT report no conflicts of interest.

## ETHICS STATMENT

Human studies and informed consent: Ethics approval for this study was granted by the Berkshire Ethics Committee (REC Ref 12/SC/0495). All applicable international, national, and/or institutional guidelines were followed. Participants consented to take part in the interview and gave copyright for their material to be used in publications.

Animal studies: No non‐human animal studies were carried out by the authors for this article.

## Data Availability

De‐identified data are available under license from the University of Oxford. All data requests should be submitted to hergadmin@phc.ox.ac.uk for consideration.
